# MicroRNAs in Metal Stress: Specific Roles or Secondary Responses?

**DOI:** 10.3390/ijms131215826

**Published:** 2012-11-27

**Authors:** Heidi Gielen, Tony Remans, Jaco Vangronsveld, Ann Cuypers

**Affiliations:** Centre for Environmental Sciences, Hasselt University, Agoralaan Building D, Diepenbeek 3590, Belgium; E-Mails: heidi.gielen@uhasselt.be (H.G.); tony.remans@uhasselt.be (T.R.); jaco.vangronsveld@uhasselt.be (J.V.)

**Keywords:** metals, oxidative stress, microRNA (miRNA), target gene

## Abstract

In plants, microRNAs (miRNAs) control various biological processes by negatively regulating the expression of complementary target genes, either (1) post-transcriptionally by cleavage or translational inhibition of target mRNA, or (2) transcriptionally by methylation of target DNA. Besides their role in developmental processes, miRNAs are main players in stress responses, including metal stress responses. Exposure of plants to excess metal concentrations disturbs the cellular redox balance and enhances ROS accumulation, eventually leading to oxidative damage or signaling. Plants modify their gene expression by the activity of miRNAs in response to metal toxicity to regulate (1) complexation of excess metals, (2) defense against oxidative stress and (3) signal transduction for controlling various biological responses. This review focuses on the biogenesis, working mechanisms and functioning of miRNAs in plants. In a final part, our current knowledge on the regulatory roles of miRNAs in plant metal stress responses is highlighted, and whether stress-regulated miRNAs have specific roles or are secondary consequences is discussed.

## 1. Introduction

Since late 19th century, metal pollution of the environment is of great concern all over the world. Due to anthropogenic activities, such as the metal industry and agriculture, concentrations of metals in soil, air and water are exceeding the natural occurrence [1,2]. Increased metal concentrations can be toxic and pose a threat to all organisms. Among these metals are essential micronutrients, like copper (Cu), zinc (Zn), iron (Fe) and manganese (Mn), which are needed for normal growth and development, but are toxic in higher amounts. In contrast, cadmium (Cd), lead (Pb), aluminum (Al) and mercury (Hg) are non-essential elements that are toxic even at low concentrations. The effects caused by metals are dose and time dependent, but also metal speciation, localization and plant developmental stage are important variables [3–5]. In plants, metals interfere with physiological processes, like water balance, mineral nutrition and photosynthesis [6,7]. At the cellular level, metals disturb the redox balance, resulting in oxidative stress [8]. Metal exposure gives rise to an increase of reactive oxygen species (ROS) via pro-oxidant stimulation or antioxidant inhibition. As a consequence, plants have to cope with metal excess and with an overproduction of ROS.

Plants use several defense mechanisms against metal stress. Initially, plants try to avoid free metal accumulation in the cells (1) by keeping the metals extracellularly through reducing the uptake and stimulating the efflux or (2) by metal complexation with ligands, such as glutathione, phytochelatins and metallothioneins [9–12]. If these defenses are insufficient to prevent the build-up of free metal ions, plants have to cope with the oxidative stress that can be directly induced by redox active metals and indirectly by non-redox active metals. Antioxidative defense mechanisms are activated to reduce elevated ROS levels and hence limit oxidative damage. They consist of metabolites, such as ascorbate, glutathione and vitamin E, as well as enzymes, like superoxide dismutases, peroxidases and catalases [13,14]. In this way, excess ROS can be scavenged and neutralized.

It is of major importance that plants respond to stresses to be able to survive and reproduce via the production of seeds. Therefore, a precise and accurate regulation of stress responses is crucial. Transcriptome analysis and proteomic studies indicate major alterations in gene expression during stress such as drought, salinity, cold and oxidative stress [15–17]. Similarly, also metal stress, such as exposure to excess Cu, Cd and Zn, leads to changes in gene expression and the activity of enzymes [14,18–20]. Gene expression is mainly determined by transcriptional activation, the half-life of the produced mRNAs and their translation efficiency. With the discovery of stress-responsive small RNAs that block specific mRNAs for translation or even cause their turn-over, post-transcriptional regulation became an important aspect of gene expression regulation during stress conditions [21]. In addition, miRNAs may direct DNA methylation of target genes and hence affect transcriptional gene regulation via epigenetic modifications [22]. Current knowledge on miRNA induced transcriptional and post-transcriptional regulation of gene expression will be reviewed, similarities or differences of metal exposure with other stresses will be discussed and progress to be made towards a better insight in understanding plant metal stress responses will be indicated.

## 2. Biogenesis and Working Mechanisms of miRNAs

### 2.1. MicroRNA Biogenesis and Incorporation in Protein Complexes

Since the discovery of small RNAs as gene regulators, the biogenesis, processing and working mechanism of these small RNAs have been intensively studied [23,24]. In plants, small RNAs are subdivided into two main categories based on their origin, namely microRNAs (miRNAs) and small interfering RNAs (siRNAs). siRNAs are derived from perfectly double-stranded RNAs that can originate from several sources, such as inverted repeats, RNA-dependent RNA polymerase (RDR) activity and RNA viruses. MicroRNAs, on the other hand, are encoded by *MIR* genes that are transcribed by RNA polymerase II (Pol II) into long RNA molecules, which form double stranded stem-loop structures [25].

**Figure 1:**
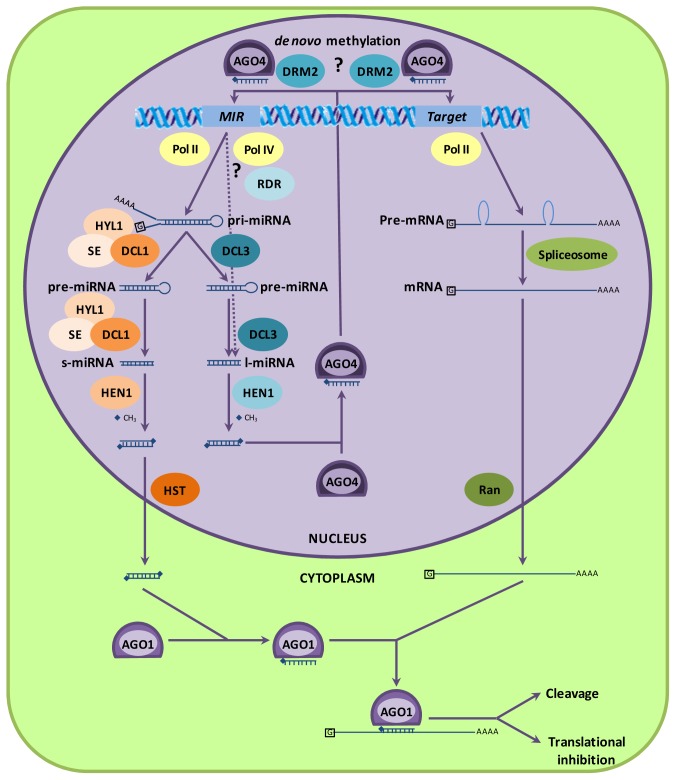
Model of the biogenesis and working mechanisms of miRNAs. *MIR* genes are transcribed by Pol II to generate a double stranded stem-loop pri-miRNA that can be further processed in short miRNAs (s-miRNAs) or long miRNAs (l-miRNAs). To produce s-miRNAs, the pri-miRNA is sliced by DCL1 and processed by HYL1 and SE. The s-miRNA/miRNA* duplex is methylated by HEN1 and exported to the cytoplasm by HST, where it associates with AGO1 into the RISC-complex. The miRNA binds with near perfect complementarity to its target mRNA to regulate it post-transcriptionally by cleavage or translational inhibition. On the other hand, to produce l-miRNAs, the pri-miRNA is sliced by DCL3 and methylated by HEN1. However, there is also the possibility that Pol IV and RDR are involved in the biogenesis of l-miRNAs. The mature l-miRNA associates with AGO4, guiding *de novo* DNA methylation probably catalyzed by DRM2. Abbreviations: miRNA gene (*MIR*), polymerase (Pol), RNA-dependent RNA polymerase (RDR), dicer-like (DCL), hyponastic leaves 1 (HYL1), serrate (SE), hua enhancer 1 (HEN1), hasty (HST), argonaute (AGO), domains rearranged methyltransferase 2 (DRM2).

Two alternative pathways to process these primary transcripts lead to miRNAs differing in both length and working mechanism (Figure 1). The best studied pathway is that of short miRNAs (s-miRNA) of 20- to 22-nt long that are sliced by dicer-like 1 (DCL1) and processed by hyponastic leaves 1 (HYL1) and serrate (SE) proteins, leading to a miRNA/miRNA* duplex that is stabilized (methylated) by hua enhancer 1 (HEN1) [26,27]. The miRNA/miRNA* duplex is exported from the nucleus to the cytoplasm by hasty (HST). Nevertheless, other export mechanisms are also present since there is no complete elimination of s-miRNAs export in *hst* knock-out mutants [28]. These mutants only showed decreased accumulation of most s-miRNAs in both cytoplasm and nucleus, suggesting the existence of an HST-independent export. The protein PAUSED (PSD), which is an Arabidopsis ortholog of the human tRNA export receptor Exportin-t, has been suggested in a possible alternative export mechanism, but no role for PSD in miRNA export could be demonstrated as miRNA accumulation in the cytoplasm was unaffected in the *psd-13* knock-out mutant [28]. In the cytoplasm, the miRNA strand (mature miRNA) is loaded into the RNA-induced silencing (RISC) complex of which Argonaute1 (AGO1) is the key component, while the miRNA* strand is usually degraded [29]. In this way, the mature s-miRNA can bind with near perfect complementarity on target mRNAs and exert its post-transcriptional regulation by cleavage of the mRNA or by translational inhibition [30,31].

Recently, another class of miRNAs was discovered with the detection of 23- to 27-nt long miRNAs (l-miRNAs) (Figure 1) [32,33]. The size classes 20- to 21-nt and 23- to 25-nt long miRNAs were filtered from publicly available Arabidopsis small RNA data sets and used in a small RNA-blot. In 16 miRNA families, only 20- to 21-nt sized miRNAs were found, 27 families consisted only of the 23- to 25-nt long miRNAs and 14 families had both size classes [32]. It is suggested that both miRNA classes derive from the same *MIR* gene and the same primary miRNA (pri-miRNA) transcript [32]. Using RNAi and knock-out mutants, the accumulation of l-miRNAs was impaired in *dcl3* mutants, but not in *dcl1*, *dcl2* and *dcl4* mutants, whereas s-miRNAs where only absent in the *dcl1* mutant [22,32,33]. Furthermore, s-miRNAs were detected in the RISC complex with AGO1 proteins as a core and were reduced in *ago1* mutants. On the other hand, AGO4 complexes all contained l-miRNAs and the accumulation of l-miRNAs in *ago4* mutants was clearly reduced [22,33]. This demonstrates that s-miRNAs are processed by DCL1 and loaded into AGO1, while l-miRNAs are sliced by DCL3 and associated with AGO4 for exerting their function (Figure 1). Since DCL3 and AGO4 are both components of the siRNA pathway, the question rises whether these l-miRNAs are also dependent of other proteins of the siRNA pathway, such as RDR2 and NRPD1 (largest subunit of Pol IV). Studies with RNAi mutants demonstrated that in rice, the processing of l-miRNAs was independent of RDR2, while in Arabidopsis, it was dependent on both RDR2 and NRPD1 [22,33]. This indicates that differences between species occur in the biogenesis pathway of l-miRNAs. Because l-miRNAs in Arabidopsis are processed by the siRNA machinery, Chellappan *et al*. [33] talk about *MIR*-derived siRNAs instead of l-miRNAs.

### 2.2. MicroRNAs Can Cause DNA Methylation

RNA-directed DNA methylation (RdDM) by AGO4-associated siRNAs induces *de novo* DNA methylation catalyzed by domains rearranged methyltransferase2 (DRM2) [34]. MicroRNA mediated DNA methylation, however, was demonstrated only in a few cases. MicroRNA165/166 was the first miRNA in Arabidopsis for which it was shown that it induces DNA methylation [35]. Its gene targets *phabulosa* (*PHB*) and *phavoluta* (*PHV*), two transcription factors that promote adaxial cell fate in the leaf primordium, are heavily methylated downstream of the complementary site. Mutations in this complementary site in *phb* and *phv* mutants resulted in reduced methylation of the respective genes [35]. Furthermore, the predicted targets of l-miR2328 and l-miR2831, *At4g16580* and *At5g08490* respectively, were shown to be methylated in an NRDP1 dependent way about ~80 nucleotides up- and downstream of the complementary site, as expression levels of the target genes were increased in the *nrdp1* mutant [33]. Wu *et al*. [22] reported hypomethylation of predicted l-miRNA targets *Os06g38480*, *Os03g02010*, *Os05g01790*, *Os07g41090* and *Os02g05890* in rice *dcl3* mutants. Moreover, the DNA methylation analysis revealed that l-miRNAs can also direct DNA methylation at their own miRNA locus in rice, whereas no change in DNA methylation was observed in Arabidopsis miRNA loci [22,33]. Altogether, these results demonstrate that l-miRNAs are processed by the siRNA machinery and exert their function by guiding DNA methylation.

The studies above demonstrate that miRNAs can cause epigenetic modifications under normal conditions. However, research has shown that also under a diverse array of biotic and abiotic stresses, DNA methylation can be modified [36–38]. Nevertheless, it has to be demonstrated whether this DNA methylation is l-miRNA dependent. *Trifolium repens* (metal-sensitive) and *Cannabis sativa* (metal-tolerant) plant species were grown on metal-contaminated soils (nickel, cadmium and chromium). DNA methylation analysis pointed out that under control conditions the genome of *Cannabis sativa* was three times more methylated than *Trifolium repens* and that the genome of both plant types was hypomethylated in a dose-dependent manner after metal stress exposure. However, the methylation level was still significantly higher in treated Cannabis plants than in control Trifolium plants [39]. This suggests that plant tolerance to excess metals can be aided by a persistent level of DNA methylation. Furthermore, salt and alkaline stress caused variations in DNA methylation of *Chloris virgata*, predominantly in the roots, and it was suggested that this may play a role in the acquirement and inheritance of salt and alkaline stress tolerance [40]. However, besides the involvement of several methyltransferases like DRM1, DRM2, DNA methyltransferase1 (MET1) and chromomethylase1 (CMT1), it is unclear how these DNA methyltransferases are targeted to genomic sites harboring genes related to stress responses. Whereas the role of epigenetic modifications in plant development is studied intensively [41–43], information on a direct link between stress, DNA methylation and l-miRNAs is scant.

### 2.3. Argonaute Proteins and miRNA Function

The dual function of *MIR* genes to regulate downstream targets (1) post-transcriptionally by mRNA cleavage or translational inhibition or (2) transcriptionally by DNA methylation, is probably correlated with the miRNA size produced and the AGO protein into which the miRNA is loaded. Arabidopsis encodes 10 AGO proteins that can be phenotypically divided into three clades [29], but the biological function of these 10 AGO proteins remains to be further elucidated [29,44]. Immunoprecipitation of AGO proteins followed by pyrosequencing of their associated small RNAs have revealed that the 5′ terminal nucleotide of a small RNA is involved in directing its AGO destination [45,46]. It has been shown that AGO1 preferentially associates with a 5′ terminal uridine (U), AGO5 with a 5′ terminal cytosine (C) and AGO4 and AGO2 with a 5′ terminal adenosine (A), while no other position within small RNAs had a bias for a particular nucleotide dependent on the associated AGO protein [45,46]. Indeed, changing the 5′ terminal nucleotide of miR391 and miR393b from a U to an A redirects it into AGO2 instead of AGO1 [45]. However, the 5′ terminal nucleotide is not the only determinant for sorting small RNAs into specific AGO proteins, since not all AGO1- and AGO4-associated small RNAs have a 5′ terminal U or A, respectively. Moreover, AGO2 and AGO4 associate preferentially with 5′ terminal A, but only a limited number of small RNAs is common in both AGOs and the types of small RNAs differs between both [45,46]. These observations imply that another sorting mechanism must exist. Wu *et al*. [22] ruled out the possibility that the size of a small RNA is a possible determinant in this process. By incubating AGO1 and AGO4 complexes with 32P-labeled 21- and 24-nt siRNAs, they demonstrated that AGO1 and AGO4 had similar binding affinities for both size classes. It is also reported that both AGO1 and AGO4 were bound to the different size classes of small RNAs [22,45,46]. The sorting of small RNAs in specific AGO proteins is through several mechanisms acting in concert in which the 5′ terminal nucleotide plays a major role and probably another signal coming from the biogenesis machinery is involved.

### 2.4. MicroRNA Biogenesis Is Regulated by a miRNA Feedback Mechanism

Because miRNA-directed regulation of gene expression is of great importance, it is almost self-evident that the miRNA biogenesis pathway itself is tightly regulated. Interestingly, the key components DCL1 and AGO1 of this pathway are regulated by a miRNA feedback mechanism. DCL1 was predicted to be a target of miR162 and in Arabidopsis *dcl1* mutants, showing reduced DCL1 protein activity, there was a high accumulation of *dcl1* RNA and a complete reduction of miR162 as compared to wild type plants [47]. Plants mutated in *Hen1*, another protein of the miRNA pathway (Figure 1), showed similar results [47]. Because the biosynthesis of miR162 was disrupted in both *dcl1* and *hen1*, miR162 accumulation was limited and the cleavage of its target DCL1 was decreased. From that, miR162 was identified to target and cleave DCL1 to fine-tune the accumulation level of DCL1 [47]. An analogous feedback mechanism for miR168 and its target AGO1 is reported [48]. MicroRNA168-resistant *ago1* mutant plants of Arabidopsis overaccumulated *ago1* mRNA and showed a decrease in miRNA levels. Furthermore, the developmental defects in the miR168-resistant *ago1* mutant plants were recovered by a compensatory synthetic miRNA that was complementary to the mutant *ago1* mRNA [48]. This demonstrates the importance of the miRNA-mediated regulation of DCL1 and AGO1 for proper functioning of the miRNA pathway. Although little is known concerning the effects of metal stress on the miRNA pathway itself, drought, salt, heat, UV-B and mechanical stress either up- or down-regulated miR162 and miR168 expression [23], indicating that the miRNA biogenesis pathway could be stress sensitive.

## 3. The Role of miRNAs in Regulating Their Downstream Targets

### 3.1. MicroRNA Functioning in Development

Discovering the function of miRNAs starts with identifying the miRNA targets, mostly at first by computational prediction [30,49], then followed by experimental validation. Methods like 5′-rapid amplification of cDNA ends (5′-RACE) and Parallel Analysis of RNA Ends (PARE) sequencing technology already identified and validated many targets of known miRNAs [50,51]. The use of knock-down, knock-out and overexpression mutants also contributed to the discovery of miRNA functions [52,53]. It is now clear that miRNAs play an important role in a large number of fundamental biological processes.

Most targets of miRNAs are transcription factors (TFs) usually having a function in plant development. In *Arabidopsis*, miR319 has been demonstrated to control leaf development by targeting teosinte branched1 cycloidea proliferating cell factor (TCP) transcription factors. Overexpression mutant plants in this *MIR* gene have crinkly leaves instead of flat leaves, and this phenomenon was rescued by the introduction of miR319-resistant TCP2 constructs [54]. Leaf polarity is regulated by the TFs phabulosa (PHB), phavulota (PHV) and revoluta (REV), all targets of miR165/166. When miR165/miR166 control of PHB, PHV and REV is suppressed, resulting in gain-of-function mutants, accumulation of these proteins is expanded from the adaxial regions to the abaxial regions in lateral organ primordia, thereby disturbing leaf polarity [55–57]. Members of the NAM/ATAF/CUC (NAC)-domain family are TFs that contain a similar N-terminal DNA-binding domain and the name of this family is derived from three proteins, namely NAM (no apical meristem), ATAF1-2 (Arabidopsis transcription activation factor) and CUC (cup-shaped cotyledon). MicroR164 targets several members of the NAC-domain family, like CUC1/2 and NAC1 [53,58]. Transgenic plants expressing a cleavage-resistant form of NAC1 mRNA had more lateral roots than the WT plants, and overexpression of miR164 had reduced NAC1 mRNA levels and less lateral roots, pointing out that the regulation of NAC1 is important for lateral root development [58]. In Arabidopsis, the regulation of CUC1 and CUC2 by miR164 constrains the expansion of the boundary domains in meristems, indicated by the observed enlarged boundary domains in miRNA-resistant CUC2 plants and in plants with reduced miRNA levels (knock-out mutants *dcl1*, *hen1* and *hyl1*) [53]. Also, members of the auxin response factor (ARF) family are regulated by miR160 and miR167, thereby regulating auxin signaling and, thus, multiple developmental responses, even reproduction [59,60]. The transitions from juvenile to vegetative to reproductive phase are controlled by squamosa promotor binding-like (SPL) family members and apetala2 (AP2)-like genes targeted by miR156 and miR172, respectively [61,62]. These few examples prove the important roles of miRNAs in development.

### 3.2. MicroRNA Functioning in Stress Responses

Although most (known) miRNAs play a role in developmental processes, evidence from the last 10 years demonstrates the involvement of miRNAs in regulating stress responses. One of the first miRNAs described to be involved in stress response is miR395 as its expression increased upon sulfate starvation (0.2 and 0.02 mM (NH_4_)_2_SO_4_) in Arabidopsis [49]. This miRNA targets two families involved in the sulfate assimilation pathway, namely ATP sulfurylases (APS) and sulfate transporter 2;1 (SULTR2;1) [49,63,64]. Overexpression of miR395 resulted in a reduction of the target transcripts and an overaccumulation of sulfate in the shoots. The RNAi triple repressed mutant *aps1*/*sultr2; 1*/*aps4* phenocopied this miR395-overexpressing mutant [65]. Also during phosphate starvation, miRNAs come into play. MicroRNA399 is up-regulated during phosphate starvation, thereby regulating Pi homeostasis by targeting phosphate2 (PHO2), an ubiquitin-conjugating E2 enzyme [66,67]. Overexpression of miR399 reduced PHO2 transcripts and the phenotype of the miR399 overexpressor was the same as *pho2* knock-out mutants. Furthermore, the remobilization of phosphate in both mutants was almost identical, demonstrating the control of phosphate homeostasis through the regulation of PHO2 by miR399 [67]. Several other studies identified stress-regulated miRNAs and some are listed in the following. Under UV-B stress conditions, 21 miRNAs belonging to 11 miRNA families showed an up-regulated expression in Arabidopsis, implying a regulatory role for these miRNAs in a UV-B stress response [68]. Micro-array data also revealed 14 miRNAs of Arabidopsis seedlings induced by high-salinity (300 mM NaCl), drought (200 mM mannitol) and low temperature (4 °C), of which miR168, miR171 and miR396 responded to all three stresses [69]. By constructing a library of small RNAs from Arabidopsis, Sunkar and Zhu [70] also found several miRNAs that were up- or down-regulated by cold (0 °C for 24 h), dehydration (for 10 h), NaCl (300 mM for 5 h) and ABA (100 μM for 3 h) treatments. These results indicate significant roles for miRNAs in responses to environmental stresses, although the primary effects on stress tolerance remain to be elucidated for the specific miRNAs.

## 4. The Role of miRNAs in Metal Stress

**Figure 2:**
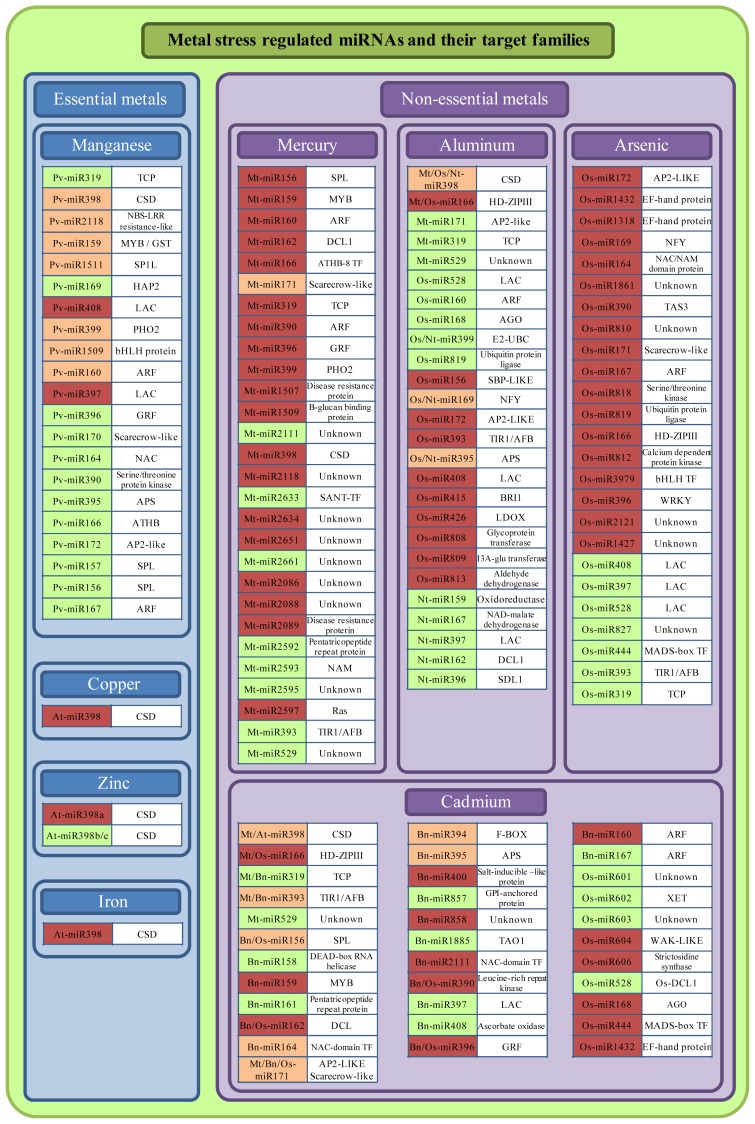
Summary of metal-regulated miRNAs in plants and their target families. MicroRNAs are categorized based on the metal they respond to. The effects of excess essential (Cu, Mn, Zn and Fe; blue boxes) and non-essential (Cd, Al, Hg and As; purple boxes) metals on the expression of miRNAs is shown (green: up-regulation; red: down-regulation; orange: up-regulation or down-regulation through contrasting results in various studies.). These metal-regulated miRNAs are obtained from several plant species, like *Arabidopsis thaliana* (At), *Phaseolus vulgaris* (Pv), *Medicago truncatula* (Mt), *Brassica napus* (Bn), *Oryza sativa* (Os) and *Nicotiana tabacum* (Nt) [14,21,71–82].

Although miRNAs have been intensively studied over the last years, little research is performed on the role of miRNAs in metal stress responses. Nevertheless, a number of studies demonstrated the involvement of miRNAs in responses to different metal toxicities, mostly using screenings like microarrays and deep sequencing of small RNA libraries. These studies were performed on different species such as *Arabidopsis thaliana*, *Medicago truncatula*, *Brassica napus*, *Oryza sativa*, *Nicotiana tabacum* and *Phaseolus vulgaris*. Plants were exposed to different metal treatments, including essential elements (Cu, Fe, Zn and Mn) and non-essential elements (Cd, Hg, Al and As). The metal-regulated miRNAs of these studies are summarized in Figure 2.

Plants initiate three major actions in response to metal toxicity, namely complexation of excess metals, defense against metal-induced oxidative stress and signal transduction for controlling various biological processes. The current knowledge of the involvement of miRNAs herein will be discussed in the following parts.

### 4.1. Role of miRNAs in Metal Complexation

After construction of a small RNA library of *Brassica napus* seedlings exposed to excess Cd and sulfate limitation, several stress-responsive miRNAs were reported among which miR395 [71]. Expression analyses revealed an up-regulation of miR395 under Cd exposure and sulfur deficiency. Several ATP sulfurylases (APS) and SULTR2;1 were identified as targets of miR395, but there was not always a consequent negative correlation between these targets and miR395 [63,71]. SULTR2;1 is a low affinity sulfate transporter functioning in the sulfate remobilization from mature into younger leaves, and the APS enzymes catalyze the first step in the sulfur assimilation pathway [65,82]. This pathway leads to the assimilation of sulfate into cysteine and further to the production of glutathione (GSH) [82]. Cadmium, mercury (Hg) and other metals have a high affinity for thiols, the functional group of GSH and phytochelatins (PCs) [4,83,84]. The chelation of these metals to GSH and PCs is therefore an important defense strategy against metal stress, since it prevents that free metal ions can exert their action. In Arabidopsis, an increase in PCs is observed in response to Cd treatments and mutants lacking PCs are hypersensitive to Cd [11,85]. Because Cd-induced miR395 regulates the sulfate assimilation pathway, and hence, indirectly the GSH and PC biosynthesis, miRNAs can have a role in the complexation of free metal ions. Furthermore, PC synthesis is up-regulated upon Zn and Hg exposure, but if this induction is regulated by miR395 remains to be proven [20,83].

Nevertheless, various regulation patterns were reported on the miR395 expression in response to excess metals. In 14 days old *Brassica napus* plants, RT-PCR analysis showed an up-regulation of miR395 at time point 72 h upon treatment with 80 μM Cd in a kinetic (12, 24, 48 and 72 h) study [71]. On the other hand, in another study with the same plant and growth conditions, sequence reads of control and Cd-treated (80 μM for 6, 24 and 48 h) miRNA libraries were compared and a down-regulation of miR395 was reported [72]. A possible explanation for these contrasting results may be found in the experimental design. The 72 h induction time point of the first study was absent in the second study and, moreover, in the latter, the samples of the same conditions, but different time points were pooled to construct the miRNA libraries, thereby excluding possible kinetic regulations of miR395. In *Medicago truncatula*, the regulation of miR395 after metal exposure was also reported. Four-day old seedlings were exposed for 6, 12, 24 or 48 h to 10 μM Hg, and an induction of miR395 was observed as determined by sequence analysis of miRNA libraries [73]. However, no response of miR395 was observed in 16 days old *Medicago truncatula* plants upon exposure to 20 μM Hg, 80 μM Cd or 50 μM Al for 24 h, analyzed with RT-PCR [74]. The differences in responses observed may be due to the different developmental stage and the exposure times of the plants in the above studies. This indicates that the miRNA response can be time-dependent, and hence, kinetic studies of miRNA expression levels may be more informative to compare responses within and between species.

A direct correlation between metal stress, miRNAs and complexation remains rather uncertain. There is a possibility that metals induce PC synthesis independently of miR395, thereby consuming sulfate into the polypeptide chain. This, in turn, causes a sulfate limitation response and, thus, an induction of miR395 for regulating the sulfate assimilation pathway. Additional experiments are needed to clarify this interconnection.

### 4.2. Role in Oxidative Stress

Toxic metal concentrations disturb the redox-balance and generate excessive amounts of ROS leading to oxidative stress [8]. These metals can be essential or non-essential elements for plants to finish their life cycle. In the following section, the role of metal-induced miRNAs in the oxidative challenge is discussed, whereby first the responses of essential metals and secondly of non-essential metals are described.

Exposure of *Arabidopsis thaliana* seedlings to excess Cu decreased miR398 transcription, resulting in an up-regulation of its targets CSD1 and CSD2, two Cu/Zn superoxide dismutases [14,21]. Redox-active metals like Cu induce oxidative stress directly via the Fenton and Haber-Weiss reactions. Therefore, an up-regulation of anti-oxidants, such as CSDs, is important for scavenging ROS and reducing damage. In *Arabidopsis*, the miR398 family consists of three loci, namely miR398a, miR398b and miR398c. The expression of all three miR398s was down-regulated when exposed to excess Cu [14,21]. Conversely, under Cu-deficiency miR398 is induced causing down-regulation of CSD1 and CSD2 [86,87]. *FSD1* (FeSOD) is up-regulated and takes over the superoxide dismutase function [86–88]. This is a coordinated response in which squamosa promoter binding protein-like7 (SPL7) binds directly to GTAC motifs in both *FSD1* and miR398b/c promoters, thereby up-regulating their expression, which causes a positive regulation of FSDs and a negative regulation of CSDs. Additionally, GTAC motifs are present in the promoters of miR397, miR408 and miR857 [87]. All these miRNAs were up-regulated during Cu deficiency, and their targets are all Cu-containing proteins [87,89]. As a result, the limited Cu is then not targeted to these proteins, but preferentially to plastocyanin, which is not miRNA regulated and is essential for photosynthesis.

Plants exposed to toxic concentrations of Fe, another essential micronutrient, and methylviologen (MV), both directly inducing oxidative stress, showed similar results as observed after excess Cu treatment. Seedlings from *Arabidopsis thaliana* were 8 or 24 h exposed to 10 μM MV or 100 μM Fe^3+^ and an expression analysis of miR398 and *CSDs* was performed [21]. MV and Fe^3+^ led to a down-regulation of miR398 and an induction of *CSD1* and *CSD2,* probably for defense against the oxidative stress. In *Arabidopsis thaliana*, the expression profiles of primary transcripts of miR398 were also analyzed after exposure to excess Zn (100, 250 or 500 μM Zn) [20]. In leaves, transcripts of miR398b/c genes showed an induced expression, while there was no difference in expression in the roots. Noteworthy, the three miR398 genes were differently regulated by Zn toxicity, which had not been reported before during stress treatment. Transcription of miR398a decreased in leaves and roots, whereas miR398b and miR398c transcript levels were induced in leaves, but showed no response in roots [20]. The authors suggested the possibility that, under Zn stress, CSD1 in the leaves is only regulated by miR398a and not by miR398b/c, since CSD1 is up-regulated. In contrast, CSD2 is down-regulated in leaves after Zn treatment, which is in accordance to the induction of miR398b/c expression.

MiR398 has also been studied in other species than Arabidopsis and upon exposure to non-essential metals. Using miRNA macroarrays, miRNAs in leaves, roots and nodules of *Phaseolus vulgaris* were detected under normal and various stress conditions (low pH (5,5); deficiency of phosphorus (P), Fe or nitrogen (N); manganese (Mn) toxicity) [75]. Manganese toxicity (200 μM) regulated 33 miRNAs in total in the three organs of which several miRNA expressions where organ specific, among which the expression of miR398. In the leaves, miR398 was decreased, whereas an induction of miR398 was observed in the roots and nodules [75]. The exposure of *Medicago truncatula* (4 or 16 days old) to non-essential metals, like 80 μM Cd, 10 or 20 μM Hg and 50 μM Al, for 6, 12, 24 or 48 h led to a decreased miR398 expression [73,74]. On the other hand, miR398 induction was reported in 21 days old *Nicotiana tabacum* seedlings after exposure to Al oxide nanoparticles from germination onwards and in 21 days old *Arabidopsis thaliana* seedlings upon treatment with 5 or 10 μM Cd for 24 h accompanied with a reduced CSD level [14,76]. A possible explanation for these results can be that non-essential metals interfere with the uptake and/or subcellular speciation and localization of essential metal micronutrients, which then influences miRNA expression as a secondary effect.

These above results may indicate that the experimental design is an important factor in the observed plant responses to metal stress. In general, metals induce oxidative stress, directly or indirectly, but the oxidative stress signature (e.g., the speciation, duration and level of ROS production, the cellular redox state…) may be dependent on the metal speciation, concentration and exposure time. For example, through down-regulation of miR398 with subsequent induction of CSD levels, plants are capable of increasing the capacity of scavenging superoxide radicals and reacting to the oxidative stress induced by excess metals. However, this response is not always straightforwardly followed by the plants (see examples above). So, it seems that different metals, exposure time, exposure concentrations, species, organ, developmental stage and cultivation techniques may influence regulation of miRNA expression, as well as regulation of the targets, and that direct effects are not always easily distinguishable from secondary effects.

### 4.3. Role in Signal Transduction

Between the perception of metal stress and the onset of cellular responses, a cascade of signaling events takes place. Metal stress induces elevated levels of ROS that may result in damage. But on the other hand, the produced ROS can function as signaling molecules, e.g., in activating MAPK pathways, to control stress responses. Several studies reported the involvement of mitogen-activated protein kinase (MAPK) pathways in metal stress signaling. In *Arabidopsis thaliana*, exposure to 2 μM Cu or 5 μM Cd resulted in the up-regulation of MPK3 and MPK6 transcript levels [90]. In addition, in *Medicago sativa,* four MAPKs (SIMK, MMK2, MMK3 and SAMK) were activated upon treatment with increasing concentrations (1, 10, 50, 100, 500 and 1000 μM) of Cu or Cd in a dose-dependent manner [91]. Furthermore, *Oryza sativa* exposed to 50 μM arsenite (As) showed increased transcript levels of OsMPK3 and OsMKK4 in leaves and roots [92]. A link between MAPK pathways and miRNAs in metal stress response was shown for OXI1 (oxidative signal inducible kinase), a component of the MAPK pathway, that is involved in the regulation of miR398b/c upon 5 μM Cd and 2 μM Cu treatment in *Arabidopsis thaliana* (ecotype Wassilewskija) seedlings. MicroRNA398b/c was up-regulated in *oxi1* knock-out mutants after Cd exposure, while in the wildtype (WT) there was no response. Treatment with Cu down-regulated the expression of miR398b/c in the WT, which was not seen in *oxi1*[93].

The final kinase in the MAPK cascade activates TFs that further downstream regulate gene expression. In addition, several TFs are targets of metal-regulated miRNAs. The targets of miR156/157 are TFs of the SPL family and play diverse roles in amongst others phase transition, flower development and plant architecture [49,72,94]. MicroRNA156/157 was down-regulated after exposure to 80 μM Cd in *Brassica napus*, 10 μM Hg in *Medicago truncatula* and 450 μM Al in rice, while it was induced upon 200 μM Mn in *Phaseolus vulgaris*[73,75,77,78].

Furthermore, several metal-induced miRNAs have targets involved in the hormone biogenesis and signaling, often via influencing transcription factors. Phytohormones, like ethylene, auxin and jasmonic acid (JA), are important signaling molecules whose production can be influenced by metal toxicity and thereby affecting signal transduction [95–98]. Several transcription factors of the TCP family are targets of miR319. The TCP TFs play a role in leaf development (cfr. supra) and hormone signaling [54,99]. In Arabidopsis, expression analyses of plants with increased activity of miR319 showed altered expression of jasmonic acid biosynthesis genes and changed levels in JA [99]. Several studies reported the regulation of miR319 under various metal stresses. The induction of miR319 was shown upon treatment with 80 μM Cd or 20 μM Hg in *Medicago truncatula* and 200 μM Mn in *Phaseolus vulgaris*, whereas it was down-regulated after treatment to 80 μM Cd in *Brassica napus* and 10 μM Hg in *Medicago truncatula*[72–75]. Another miRNA involved in hormone signaling is miR171, which targets scarecrow-like (SCL) transcription factors that function in a wide range of developmental processes, including radial patterning in roots and hormone signaling [100]. An *scl3* null mutant displayed reduced gibberellin (GA) responses and an induced expression of GA biosynthesis genes, indicating that SCL3 can positively regulate GA signaling [101]. MicroRNA171 was down-regulated after 40, 60 or 80 μM Cd exposure in *Brassica napus* and *Oryza sativa* and 10 μM Hg exposure in *Medicago truncatula*[72,73,77,79], whereas Zhou *et al*. [74] reported the induction of miR171 after exposure to 80 μM Cd, 20 μM Hg or 50 μM Al in *Medicago truncatula*. MicroRNAs are also involved in the auxin signaling. In *Arabidopsis*, by using 5′-RACE analysis, it was detected that miR393 guides the cleavage of the 4 auxin receptors transport inhibitor response1 (TIR1), auxin signaling F-BOX1 (AFB1), AFB2 and AFB3. Real-time RT-PCR showed that these targets of miR393 were all up-regulated in the leaves of the T-DNA insertion mutants, *mir393b* and *dcl1-9*. Furthermore, the *mir393b* mutant plants compared to the WT showed a greater number of leaves, more leaf elongation and more leaf epinasty, a typical phenotype for auxin hypersensitivity [102]. These results indicate that miR393 is involved in auxin signaling by regulating its four auxin receptor targets. Moreover, miR393 is identified to be metal-responsive, since miR393 expression was up-regulated in *Medicago truncatula* upon 80 μM Cd and 20 μM Hg, and decreased upon 80 μM Cd in *Brassica napus* and 450 μM Al in rice [74,77,78].

Altogether, it is clear that metal stress-induced signal transduction involves a multitude of signaling components that interact with each other and whereby miRNAs play an important role that needs to be taken into account in future studies.

### 4.4. Other Metal Stress Regulated miRNAs

Besides the metal stress regulated miRNAs discussed in the above parts, a number of studies demonstrated also the regulation of non-conserved species-specific miRNAs under metal stress (see Figure 2). The function of these miRNAs is mostly unknown, since the targets of these miRNAs are unknown proteins, unspecific TFs or have an unspecific role in metabolism. In addition, several conserved miRNAs that have no (known) function in metal complexation, oxidative stress or signal transduction were identified to be metal stress regulated, like miR397 and miR408 (see Figure 2) [72,75,76,78,80]. Both miRNAs target laccases (LAC) that are Cu-containing proteins able to catalyze the oxidation of various substrates, such as phenols and amines [103]. It is suggested that these enzymes are involved in lignin biosynthesis, but the definitive function remains largely unknown [103,104]. In Arabidopsis, T-DNA insertion mutations in *LAC4* and *LAC17* resulted in reduced stem lignifications, demonstrating that these laccases have a function in lignin synthesis [105]. Several studies reported an increased lignin synthesis upon metal treatment [106,107]. Furthermore, X-ray spectroscopy analysis in *Juglans regia* demonstrated that metal ions (Pb) can interact with the oxygen atoms of lignin, providing evidence that lignins can complex metal ions [106]. These results suggest a role for miR397 and miR408 in extracellular metal complexation by targeting laccases, but this function remains to be elucidated.

## 5. Stress-Specific Regulation of miRNAs Is Not Always Straightforwardly Connected to Beneficial Target Gene Regulation

Plants have to react in a correct way to various stresses to be able to complete their life cycle. Different stresses determine a specific regulation of the stress response. Also microRNA expression is differently regulated by several kinds of stresses. In *Arabidopsis thaliana*, UV-B radiation, cold stress and salt stress induced expression of miR169, while it was down-regulated by ABA and drought stress [23]. Also, for miR395, a diverse expression array was observed, being induced by sulfate deprivation and down-regulated by hypoxia. Interestingly, the same kind of stress can induce contrasting miRNA responses in different species. For example, upon salt stress miR167 was induced in *Arabidopsis thaliana,* but down-regulated in *Zea mays*[23]. Furthermore, different metals can provoke different responses of miRNA expression. For example, miR398 is up-regulated by Cu excess, but the expression decreased after Cd treatment [14]. Moreover, miRNAs of the same family can also be differently regulated, as seen after exposure to excess Zn, where miR398a transcript levels were decreased and levels of miR398b and miR398c were induced [20]. These examples indicate that the regulation of miRNAs is stress-specific and clearly emphasizes the need for further research to a gain better insight in these regulations. Genetic screens using mutagenized transgenic miRNA promoter-reporter lines may be used to identify upstream events leading to up- or down-regulation of miRNA promoter activity.

Activation of a *MIR* gene to form pri-miRNA transcripts, processing of these into a mature miRNA, and subsequent down-regulation of the mRNA target, is a sequence of events that is sometimes followed straightforwardly. It is interesting to argue that this scheme of regulation is followed strictly when the miRNA-mediated regulation of the target mRNA is essential and functionally important. Hence, it has evolved evolutionary as a dominant and direct response leading to correct regulation of target gene expression, for example, during development. Deviations can occur in this process, for example, a “non-logical” response or an absence of correspondence between miRNA expression and target regulation. These deviations may be the result of a less dominant regulation by miRNA and the involvement of other regulations, for example, at the transcriptional level. This is often observed as the consequence of stress effects and lead to secondary responses that are either beneficial, neutral or disadvantageous responses.

Cd exposure leads to oxidative stress, and exposure to various agents causing oxidative stress have been reported to down-regulate miR398, thereby allowing increased CSD expression levels [21]. Strikingly, a “non-logical” response was observed for miR398, which was up-regulated thereby down-regulating CSDs after Cd treatment [14]. This may be a secondary response that is not necessarily beneficial for the plant.

A large number of miRNAs have different targets that do not necessarily belong to the same gene family. Interestingly, not all targets of a miRNA are regulated in the same way. While miR395 was induced upon sulfate deficiency, its APS targets were not all down-regulated. *APS1* showed no response, *APS3* was up-regulated, while *APS4* was induced after sulfate deficiency [65].

Another possible deviation of the miRNA-target process is that both miRNA and target are regulated in the same way. In this case, a certain stress stimulates the transcription of a miRNA and its target. The outcome of this response is dependent of the balance between the transcription rate and the post-transcriptional regulation of the target. Several studies have already investigated the proteins involved in the miRNA biogenesis pathway and the RISC complex, but additional protein factors, e.g., that may cause stress specific regulations, cannot be ruled out. There may be some (unknown) protein factors of the RISC complex, some of which are perhaps stress-dependent, that may determine whether miRNA-target interaction takes place. It is clear that the elucidation of the exact role of miRNAs in regulating stress responses has a complexity that in many cases remains to be untied, as it cannot be explained by all current knowledge on mechanisms of miRNA action.

## 6. Conclusions

A precise and accurate regulation of stress responses is of major importance for plants to be able to complete their life cycle. Upon metal stress, major alterations in the gene expression of plants occur to regulate complexation of the metals, defense against the metal-induced oxidative stress and changes in various biological processes. MicroRNAs are crucial components of the gene regulatory network through their negative regulation of target genes. A complete insight into the functions of miRNAs will increase our understanding of plant responses to metal stress. Therefore, the identification of entire sets of metal-regulated miRNAs and their targets in a tissue-specific manner is needed. If these identified metal-regulated miRNAs are specifically altered in their gene expression for adjustment and tolerance to the metal stress or if these alterations in miRNA expression are secondary consequences of a disturbed cellular homeostasis due to the metal stress, remains to be uncovered in future studies.

## References

[1] Nriagu J.O., Pacyna J.M. (1988). Quantitative assessment of worldwide contamination of air, water and soils by trace metals. Nature.

[2] Vangronsveld J., van Assche F., Clijsters H. (1995). Reclamation of a bare industrial area contaminated by non-ferrous metals: in situ metal immobilization and revegetation. Environ. Pollut.

[3] Keunen E., Truyens S., Bruckers L., Remans T., Vangronsveld J., Cuypers A. (2011). Survival of Cd-exposed *Arabidopsis thaliana*: Are these plants reproductively challenged?. Plant Physiol. Biochem.

[4] Verbruggen N., Hermans C., Schat H. (2009). Mechanisms to cope with arsenic or cadmium excess in plants. Curr. Opin. Plant Boil.

[5] Lahive E., O’Halloran J., Jansen M.A.K. (2012). Frond development gradients are determinant of the impact of zinc on photosynthesis in three species of *Lemnaceae*. Aquat. Bot.

[6] Dalcorso G., Farinati S., Maistri S., Furini A. (2008). How plants cope with cadmium: Staking all on metabolism and gene expression. J. Integr. Plant Biol.

[7] Yruela I. (2009). Copper in plants: Acquisition, transport and interactions. Funct. Plant Biol.

[8] Sharma S.S., Dietz K.J. (2009). The relationship between metal toxicity and cellular redox imbalance. Trends Plant Sci.

[9] Hall J.L. (2002). Cellular mechanisms for heavy metal detoxification and tolerance. J. Exp. Bot.

[10] Zhang H.Y., Xu W.Z., Guo J.B., He Z.Y., Ma M. (2005). Coordinated responses of phytochelatins and metallothioneins to heavy metals in garlic seedlings. Plant Sci.

[11] Semane B, Dupae J., Cuypers A., Noben J.P., Tuomainen M., Tervahauta A., Kärenlampi S., van Belleghem F., Smeets K., Vangronsveld J. (2007). Leaf proteome responses of *Arabidopsis thaliana* exposed to mild cadmium stress. J. Plant Physiol..

[12] Seth C.S., Remans T., Keunen E., Jozefczak M., Gielen H., Opdenakker K., Weyens N., Vangronsveld J., Cuypers A. (2012). Phytoextraction of toxic metals: A central role for glutathione. Plant Cell Environ.

[13] Smeets K., Opdenakker K., Remans T., van Sanden S., van Belleghem F., Semane B., Horemans N., Guisez Y., Vangronsveld J., Cuypers A. (2009). Oxidative stress-related responses at transcriptional and enzymatic levels after exposure to Cd or Cu in a multipollution context. J. Plant Physiol.

[14] Cuypers A., Smeets K., Ruytinx J., Opdenakker K., Keunen E., Remans T., Horemans N., Vanhoudt N., van Sanden S., van Belleghem F. (2011). The cellular redox state as a modulator in cadmium and copper responses in *Arabidopsis thaliana* seedlings. J. Plant Physiol.

[15] Seki M., Narusaka M., Ishida J., Nanjo T., Fujita M., Oono Y., Kamiya A., Nakajima M., Enju A., Sakurai T. (2002). Monitoring the expression profiles of 7000 *Arabidopsis* genes under drought, cold and high-salinity stresses using a full-length cDNA microarray. Plant J.

[16] Shinozaki K., Yamaguch-Shinozaki K. (2007). Gene networks involved in drought stress response and tolerance. J. Exp. Bot.

[17] Dos Reis S.P., Lima A.M., de Souza C.R.B. (2012). Recent molecular advances on downstream plant responses to abiotic stress. Int. J. Mol. Sci.

[18] Zhang H., Zhang F., Xia Y., Wang G., Chen Z. (2010). Excess copper induces production of hydrogen peroxide in the leaf of *Elsholtzia haichowensis* through apoplastic and symplastic CuZn-superoxide dismutase. J. Hazard. Mater.

[19] Drazkiewicz M., Skórzynska-Polit E., Krupa Z. (2010). Effect of BSO-supplemented heavy metals on atioxidant enzymes in *Arabidopsis thaliana*. Ecotoxicol. Environ. Saf.

[20] Remans T., Opdenakker K., Guisez Y., Carleer R., Schat H., Vangronsveld J., Cuypers A. (2012). Exposure of *Arabidopsis thaliana* to excess Zn reveals a Zn-specific oxidative stress signature. Environ. Exp. Bot.

[21] Sunkar R., Kapoor A., Zhu J.K. (2006). Posttranscriptional induction of two Cu/Zn superoxide dismutase genes in *Arabidopsis* is mediated by downregulation of miR398 and important for oxidative stress tolerance. Plant Cell.

[22] Wu L, Zhou H., Zhang Q., Zhang J., Ni F., Liu C., Qi Y. (2010). DNA methylation mediated by a microRNA pathway. Mol. Cell.

[23] Khraiwesh B., Zhu J.K., Zhu J. (2012). Role of miRNAs and siRNAs in biotic and abiotic stress responses of plants. Biochim. Biophys. Acta.

[24] Jamalkandi S.A., Masoudi-Nejad A. (2009). Reconstruction of *Arabidopsis thaliana* fully integrated small RNA pathway. Funct. Integr. Genomics.

[25] Lee Y., Kim M., Han J., Yeom K.H., Lee S., Baek S.H., Kim V.N. (2004). MicroRNA genes are transcribed by polymerase II. EMBO J.

[26] Yu B., Yang Z., Li J., Minakhina S., Yang M., Padgett R.W., Steward R., Chen X. (2005). Methylation as a crucial step in plant miRNA biogenesis. Science.

[27] Kurihara Y., Takashi Y., Watanabe Y. (2006). The interaction between DCL1 and HYL1 is important for efficient and precise processing of pri-miRNA in plant microRNA biogenesis. RNA.

[28] Park M.Y., Wu G., Gonzalez-Sulser A., Vaucheret H., Poethig R.S. (2005). Nuclear processing and export of microRNAs in *Arabidopsis*. Proc. Natl. Acad. Sci. USA.

[29] Vaucheret H. (2008). Plant argonautes. Trends Plant Sci.

[30] Rhoades M.W., Reinhart B.J., Lim L.P., Burge C.B., Bartel B., Bartel D.P. (2002). Prediction of plant miRNA targets. Cell.

[31] Mallory A.C., Bouché N. (2008). MicroRNA-directed regulation: To cleave or not to cleave. Trends Plant Sci.

[32] Vazquez F., Blevins T., Ailhas J., Boller T., Meins F. (2008). Evolution of *Arabidopsis MIR* genes generates novel microRNA classes. Nucleic Acids Res..

[33] Chellappan P., Xia J., Zhou X., Gao S., Zhang X., Coutino G., Vazquez F., Zhang W., Jin H. (2010). siRNAs from miRNA sites mediate DNA methylation of target genes. Nucleic Acids Res.

[34] Matzke M., Kanno T., Daxinger L., Huettel B., Matzke A.J. (2009). RNA-mediated chromatin-based silencing in plants. Curr. Opin. Cell Biol.

[35] Bao N., Lye K.W., Barton M.K. (2004). MicroRNA binding sites in *Arabidopsis* class III HD-ZIP mRNAs are required for methylation of the template chromosome. Dev. Cell.

[36] Chinnusamy V., Zhu J.K. (2009). Epigenetic regulation of stress responses in plants. Curr. Opin. Plant Biol.

[37] Boyko A., Kovalchuk I. (2008). Epigenetic control of plant stress response. Environ. Mol. Mutagen.

[38] Luo M., Liu X., Singh P., Cui Y., Zimmerli L., Wu K. (2012). Chromatin modifications and remodeling in plant abiotic stress responses. Biochim. Biophys. Acta.

[39] Aina R., Sgorbati S., Santagostino A., Labra M., Ghiani A., Citterio S. (2004). Specific hypomethylation of DNA is induced by heavy metals in white clover and industrial hamp. Physiol. Plant.

[40] Cao D., Gao X., Liu J., Wang X., Geng S., Yang C., Liu B., Shi D. (2012). Root-specific DNA methylation in *Chloris virgata*, a natural alkaline-resistant halophyte, in response to salt and alkaline stresses. Plant Mol. Biol. Rep.

[41] Jiang H., Kohler C. (2012). Evolution, function and regulation of genomic imprinting in plant seed development. J. Exp. Bot.

[42] Kuhlmann M., Mette M.F. (2012). Developmentally non-redundant SET domain proteins SUVH2 and SUVH9 are required for transcriptional gene silencing in *Arabidopsis thaliana*. Plant Mol. Biol.

[43] Berr A., Shafiq S., Shen W.H. (2011). Histone modifications in transcriptional activation during plant development. BBA Gene Regul. Mech.

[44] Mallory A., Vaucheret H. (2010). Form, function and regulation of ARGONAUTE proteins. Plant Cell.

[45] Mi S., Cai T., Hu Y., Chen Y., Hodges E., Ni F., Wu L., Li S., Zhou H., Long C. (2008). Sorting of small RNAs into *Arabidopsis* Argonaute complexes is directed by the 5′ terminal nucleotide. Cell.

[46] Wang H., Zhang X., Liu J., Kiba T., Woo J., Ojo T., Hafner M., Tuschl T., Chua N.H., Wang X.J. (2011). Deep sequencing of small RNAs specifically associated with *Arabidopsis* AGO1 and AGO4 uncovers new AGO functions. Plant J.

[47] Xie Z., Kasschau K.D., Carrington J.C. (2003). Negative feedback regulation of *Dicer-like1* in *Arabidopsis* by miRNA-guided mRNA degradation. Curr. Biol.

[48] Vaucheret H., Vazquez F., Crété P., Bartel D.P. (2004). The action of *ARGONAUTE1* in the miRNA pathway and its regulation by the miRNA pathway are crucial for plant development. Gene Dev.

[49] Jones-Rhoades M.W., Bartel D.P. (2004). Computational identification of plant microRNAs and their targets, including a stress-induced miRNA. Mol. Cell.

[50] Zhang B., Pan X., Wang Q., Cobb G.P., Anderson T.A. (2006). Computational identification of miRNAs and their targets. Comput. Biol. Chem.

[51] Addo-Quaye C., Eshoo T.F., Bartel D.P., Axtell M.J. (2008). Endogenous siRNA and miRNA targets identified by sequencing of the *Arabidopsis* degradome. Curr. Biol.

[52] Liu B., Li P.C., Li X., Liu C.Y., Cao S.Y., Chu C.C., Cao X.F. (2005). Loss of function of *OsDCL1* affects miRNA accumulation and causes developmental defects in rice. Plant Physiol.

[53] Laufs P., Peaucelle A., Morin H., Traas J. (2004). MicroRNA regulation of the CUC genes is required for boundary size control in *Arabidopsis* meristems. Development.

[54] Palatnik J.F., Allen E., Wu X., Schommer C., Schwab R., Carrington J.C., Weigel D. (2003). Control of leaf morphogenesis by microRNAs. Nature.

[55] Emery J.F., Floyd S.K., Alvarez J., Eshed Y., Hawker N.P., Izhaki A., Baum S.F., Bowman J.L. (2003). Radical patterning of *Arabidopsis* shoots by class III HD-ZIP and KANADI genes. Curr. Biol.

[56] Mallory A.C., Reinhart B.J., Jones-Rhoades M.W., Tang G., Zamore P.D., Kathryn Barton M., Bartel D.P. (2004). MicroRNA control of *PHABULOSA* in leaf development: Importance of pairing to the microRNA 5′ region. EMBO J.

[57] Williams L., Grigg S.P., Xie M., Christensen S., Fletcher J.C. (2005). Regulation of *Arabidopsis* shoot apical meristem and lateral organ formation by microRNA *miR166g* and its *AtHD*-*ZIP* target genes. Development.

[58] Guo H.S., Xie Q., Fei J.F., Chua N.H. (2005). MicroRNA directs mRNA cleavage of the transcription factor *NAC1* to downregulate auxin signals for *Arabidopsis* lateral root development. Plant Cell.

[59] Mallory A.C., Bartel D.P., Bartel B. (2005). MicroRNA-directed regulation of *Arabidopsis AUXIN RESPONSE FACTOR17* is essential for proper development and modulates expression of early auxin response genes. Plant Cell.

[60] Wu M.F., Tian Q., Reed J.W. (2006). *Arabidopsis microRNA167* controls patterns of *ARF6* and *ARF8* expression, and regulates both female and male reproduction. Development.

[61] Wu G., Poethig R.S. (2006). Temporal regulation of shoot development in *Arabidopsis thaliana* by *miR156* and its target *SPL3*. Development.

[62] Lauter N., Kampani A., Carlson S., Goebel M., Moose S.P. (2005). microRNA172 down-regulates glossy15 to promote vegetative phase change in maize. Proc. Natl. Acad. Sci. USA.

[63] Allen E., Xie Z., Gustafson A.M., Carrington J.C. (2005). microRNA-directed phasing during *trans*-acting siRNA biogenesis in plants. Cell.

[64] Kawashima C.G., Yoshimoto M., Maruyama-Nakashita A., Tsuchiya Y.N., Saito K., Takahashi H., Dalmay T. (2009). Sulphur starvation induces the expression of microRNA-395 and one of its targets genes but in different cell types. Plant J.

[65] Liang G., Yang F., Yu D. (2010). microRNA395 mediates regulation of sulfate accumulation and allocation in *Arabidopsis thaliana*. Plant J.

[66] Fujii H., Chiou T.J., Lin S.I., Aung K., Zhu J.K. (2005). A miRNA involved in phosphate starvation response in *Arabidopsis*. Curr. Biol.

[67] Chiou T.J., Aung K., Lin S.I., Wu C.C., Chiang S.F., Su C.I. (2006). Regulation of phosphate homeostasis by microRNA in *Arabidopsis*. Plant Cell.

[68] Zhou X., Wang G., Zhang W. (2007). UV-B responsive microRNA genes in *Arabidopsis thaliana*. Mol. Syst. Biol.

[69] Liu H.H., Tian X., Li Y.J., Wu C.A., Zheng C.C. (2008). Microarray-based analysis of stress-regulated microRNAs in *Arabidopsis thaliana*. RNA.

[70] Sunkar R., Zhu J.K. (2004). Novel and stress-regulated microRNAs and other small RNAs from *Arabidopsis*. Plant Cell.

[71] Huang S.Q., Xiang A.L., Che L.L., Chen S., Li H., Song J.B., Yang Z.M. (2010). A set of miRNAs from *Brassica napus* in response to sulphate deficiency and cadmium stress. Plant Biotechnol. J.

[72] Zhou Z.S., Song J.B., Yang Z.M. (2012). Genome-wide identification of *Brassica napus* microRNAs and their targets in response to cadmium. J. Exp. Bot.

[73] Zhou Z.S., Zeng H.Q., Liu Z.P., Yang Z.M. (2012). Genome-wide identification of *Medicago truncatula* microRNAs and their targets reveals their differential regulation by heavy metal. Plant Cell Environ.

[74] Zhou Z.S., Huang S.Q., Yang Z.M. (2008). Bioinformatic identification and expression analysis of new microRNAs from *Mecicago truncatula*. Biochem. Biophys. Res. Commun.

[75] Valdes-Lopez O., Yang S.S., Aparicio-Fabre R., Graham P.H., Reyes J.L., Vance C.P., Hernández G. (2010). MicroRNA expression profile in common bean (*Phaseolus vulgaris*) under nutrient deficiency stresses and manganese toxicity. New Phytol.

[76] Burklew C.E., Ashlock J., Winfrey W.B., Zhang B. (2012). Effects of aluminum oxide nanoparticles on the growth, development, and microRNA expression of tobacco (*Nicotiana tabacum*). PLoS One.

[77] Xie F.L., Huang S.Q., Guo K., Xiang A.L., Zhu Y.Y., Nie L., Yang Z.M. (2007). Computational identification of novel microRNAs and targets in *Brassica napus*. FEBS Lett.

[78] Lima J.C., Arenhart R.A., Margis-Pinheiro M., Margis R. (2011). Aluminum triggers broad changes in microRNA expression in rice roots. Genet. Mol. Res.

[79] Ding Y., Chen Z., Zhu C. (2011). Microarray-based analysis of cadmium-responsive microRNAs in rice (*Oryza sativa*). J. Exp. Bot.

[80] Liu Q., Zhang H. (2012). Molecular identification and analysis of arsenite stress-responsive miRNAs in rice. J. Agric. Food Chem.

[81] Huang S.Q., Peng J., Qiu C.X., Yang Z.M. (2009). Heavy metal-regulated new miRNAs from rice. J. Inorg. Biochem.

[82] Kopriva S. (2006). Regulation of sulfate assimilation in *Arabidopsis* and beyond. Ann. Bot.

[83] Carrasco-Gil L., Álvarez-Fernández A., Sobrino-Plata J., Milán R., Carpena-Ruiz R.O., Leduc D.L., Andrews J.C., Abadía J., Hernández L.E. (2011). Complexation of Hg with phytochelatins is important for plant Hg tolerance. Plant Cell Environ.

[84] Cobbett C., Goldsbrough P. (2002). Phytochelatins and metallothioneins: roles in heavy metal detoxification and homeostasis. Annu. Rev. Plant Biol.

[85] Howden R., Goldsbrough P.B., Andersen C.R., Cobbett C.S. (1995). Cadmium-sensitive, *cad1* mutants of *Arabidopsis thaliana* are phytochelatin deficient. Plant Physiol.

[86] Yamasaki H., Abdel-Ghany S.E., Bohu C.M., Kobayashi Y., Shikanai T., Pilon M. (2007). Regulation of copper homeostasis bu micro-RNA in *Arabidopsis*. J. Biol. Chem.

[87] Yamasaki H., Hayashi M., Fukazawa M., Kobayashi Y., Shikanai T. (2009). *SQUAMOSA* promoter binding protein-like7 is a central regulator for copper homeostasis in *Arabidopsis*. Plant Cell.

[88] Abdel-Ghany S.E., Burkhead J.L., Gogolin K.A., Andrés-Colás N., Bodecker J.R., Puig S., Peñarrubia L., Pilon M. (2005). AtCCS is a functional homolog of the yeast copper chaperone Ccs1/Lys7. FEBS Lett.

[89] Abdel-Ghany S.E., Pilon M. (2008). MicroRNA-mediated systemic down-regulation of copper protein expression in response to low copper availability in *Arabidopsis*. J. Biol. Chem.

[90] Opdenakker K., Remans T., Keunen E., Vangronsveld J., Cuypers A. (2012). Exposure of *Arabidopsis thaliana* to Cd or Cu excess leads to oxidative stress mediated alterations in MAPKinase transcript levels. Environ. Exp. Bot.

[91] Jonak C., Nakagami H., Hirt H. (2004). Heavy metal stress. Activation of distinct mitogen-activated protein kinase pathways by copper and cadmium. Plant Physiol.

[92] Rao K.P., Vani G., Kumar K., Wankhede D.P., Misra M., Gupta M., Sinha A.K. (2011). Arsenic stress activates MAP kinase in rice roots and leaves. Arch. Biochem. Biophys.

[93] Smeets K., Opdenakker K., Remans T., Forzani C., Hirt H., Vangronsveld J., Cuypers C (2012). The role of the kinase OXI1 in cadmium and copper induced molecular responses in*Arabidopsis thaliana*. Plant Cell Environ.

[94] Chen M., Meng Y., Mao C., Chen D., Wu P. (2010). Methodological framework for functional characterization of plant microRNAs. J. Exp. Bot.

[95] Sandmann G., Böger P. (1980). Copper-mediated lipid peroxidation processes in photosynthetic membranes. Plant Physiol.

[96] Maksymiec W., Wianowska D., Dawidowicz A.L., Radkiewicz S., Mardarowicz M., Krupa Z. (2005). The level of jasmonic acid in *Arabidopsis thaliana* and *Phaseolus coccineus* plants under heavy metal stress. J. Plant Physiol.

[97] Peto A., Lehotai N., Lozano-Juste J., León J., Tari I., Erdei L., Kolbert Z. (2011). Involvement of nitric oxide and auxin signal transduction of copper-induced morphological responses in *Arabidopsis* seedlings. Ann. Bot.

[98] Maksymiec W. (2007). Signaling responses in plants to heavy metal stress. Acta Physiol. Plant.

[99] Schommer C., Palatnik J.F., Aggarwal P., Chételat A., Cubas P., Farmer E.E., Nath U., Weigel D. (2008). Control of jasmonate biosynthesis and senescense by miR319 targets. PLoS Biol.

[100] Llave C., Kasschau K.D., Rector M.A., Carrington J.C. (2002). Endogenous and silencing-associated small RNAs in plants. Plant Cell.

[101] Zhang Z.L., Ogawa M., Fleet C.M., Zentella R., Hu J., Heo J.O., Lim J., Kamiya Y., Yamaguchi S., Sun T.P. (2011). SCARECROW-LIKE3 promotes gibberellin signaling by antagonizing master growth repressor DELLA in *Arabidopsis*. Proc. Natl. Acad. Sci. USA.

[102] Si-Ammour A., Windels D., Arn-Bouloires E., Kutter C., Ailhas J., Meins F., Vazquez F. (2011). MiR393 and secondary siRNAs regulate expression of the *TIR1/AFB2* auxin receptor clade and auxin-related development of *Arabidopsis* leaves. Plant Physiol.

[103] Turlapati P.V., Kim K.W., Davin L.B., Lewis N.G. (2011). The laccase multigene family in *Arabidopsis thaliana*: towards addressing the mystery of their gene function(s). Planta.

[104] Liang M.X., Davis E., Gardner D., Cai X.N., Wu Y.J. (2006). Involvement of AtLAC15 in lignin synthesis in seeds and in root elongation of Arabidopsis. Planta.

[105] Berthet S., Demont-Caulet N., Pollet B., Bidzinsky P., Cézard L., Le Bris P., Borrega N., Hervé J., Blondet E., Balzergue S. (2011). Disruption of *LACCASE4* and *17* results in tissue-specific alterations to lignifications of *Arabidopsis thaliana* stems. Plant Cell.

[106] Marmiroli M., Antonioli G., Maestri E., Marmiroli N. (2005). Evidence of the involvement of plant lingo-cellulosic structure in the sequestration of Pb: An X-ray spectroscopy-based analysis. Environ. Pollut.

[107] Elobeid M., Gobel C., Feussner I., Polle A. (2012). Cadmium interferes with auxin physiology and lignifications in poplar. J. Exp. Bot.

